# Efficacy and safety of antibody-drug conjugates in HER2-positive and HER2-low advanced gastric cancer: a systematic review and update

**DOI:** 10.1186/s43046-026-00388-1

**Published:** 2026-07-22

**Authors:** Lili Lei, Kun Hu, Shuangwei Xie, Bingqi Dong, Zhuona Rong, Xiaocong Pang, Junling Zhang, Ying Zhou

**Affiliations:** Peking University First Hospital, Beijing, China

**Keywords:** Advanced gastric cancer, HER2-positive, HER2-low antibody-drug conjugate, Targeted therapy, Clinical efficacy, Safety, Drug resistance mechanism

## Abstract

**Purpose:**

Gastric cancer is a highly prevalent malignancy of the digestive tract in China. Conventional chemotherapeutic drugs and human epidermal growth factor receptor 2 (HER2)‑targeted agents such as trastuzumab remain limited by significant challenges in the treatment of GC, including high rates of drug resistance, significant toxicity and adverse effects, and suboptimal tolerability. The advent of antibody-drug conjugates (ADCs) has marked a paradigm shift in the therapeutic landscape. This review systematically summarises the structural design, mechanisms of action, and current clinical applications of ADCs in HER2-positive or HER2-low advanced gastric cancer.

**Methods:**

The present review focuses on key clinical trial data for new-generation ADCs, specifically trastuzumab deruxtecan (T-DXd) and disitamab vedotin (RC48), drawing from the DESTINY-Gastric series and the RC48-C008 study. The review systematically synthesised data on efficacy, safety profiles, resistance mechanisms, and future therapeutic directions.

**Results:**

New-generation ADCs have demonstrated significant improvements in objective response rates (ORR) and overall survival (OS) compared with traditional chemotherapy in later-line treatment settings. Emerging evidence also suggests the presence of activity in HER2-low-expressing populations. A systematic assessment of adverse drug reactions highlights both common events (e.g. gastrointestinal reactions, haematologic toxicity) and distinctive adverse events (e.g. interstitial lung disease), with corresponding management strategies. A comprehensive analysis of multiple resistance mechanisms, including HER2 heterogeneity, endocytic barriers, drug efflux, and target mutations, is conducted.

**Conclusion:**

The present study demonstrates that ADCs represent a transformative therapeutic modality for HER2-positive or HER2-low cases. Ongoing advancements in ADC structural optimisation, combination strategies with immune checkpoint inhibitors show great promise in terms of further improving clinical outcomes. The objective of this review is to furnish clinicians and researchers with a detailed reference for future clinical practice and investigation.

## Introduction

**Fig. 1:**
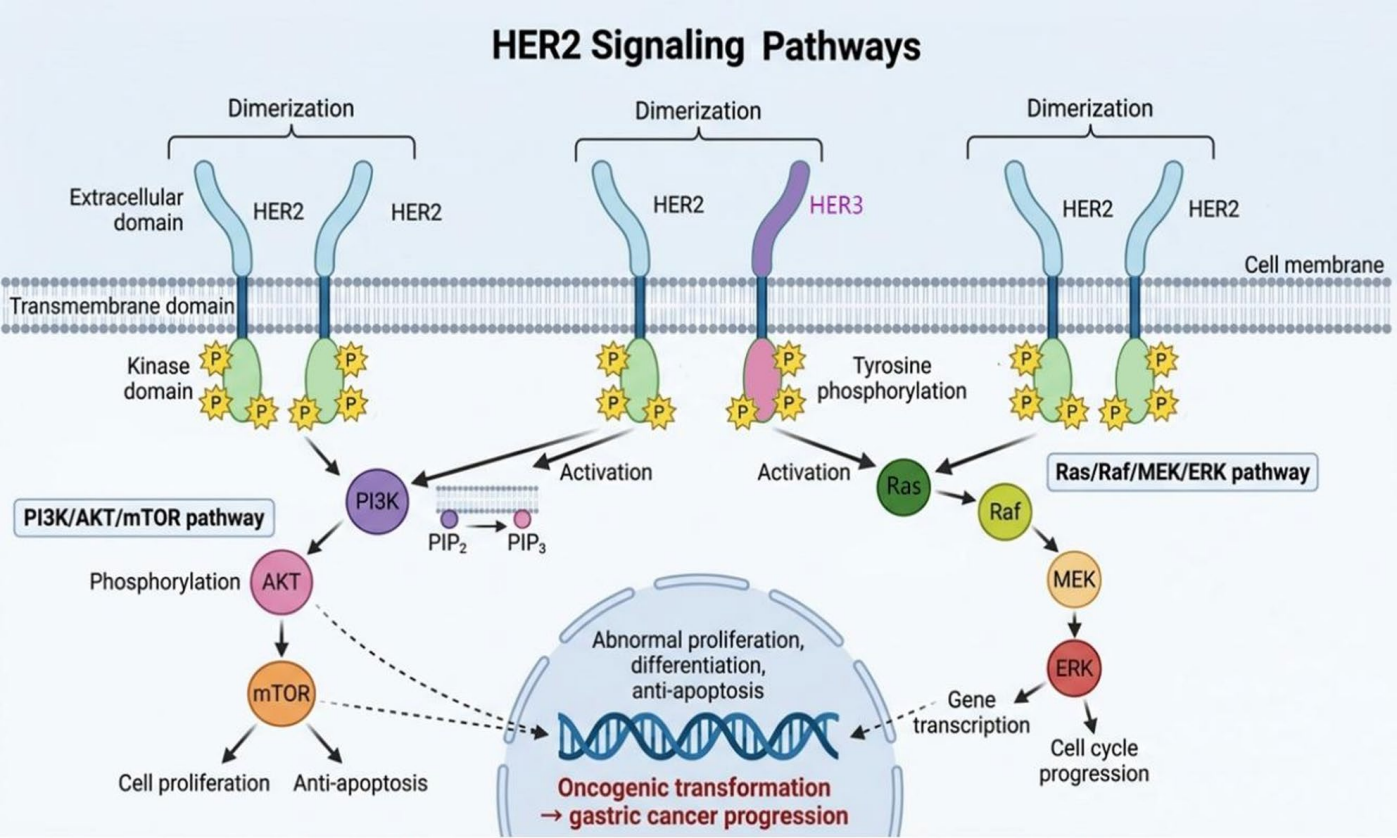
HER2 signaling pathway. The dimerization of HER2 receptor triggers two key intracellular signaling cascades-the MAPK (RAS/RAF/MEK/ERK) pathways and PI3K/AKT pathways which collectively promote cell division, proliferation, differentiation and invasive growth

Gastric cancer (GC) is the fifth most common gastrointestinal cancer in terms of both incidence and mortality on a global scale. In China, new cases and deaths account for approximately 37.1% and 39.4% of the global totals, respectively [1]. With the development of precision medicine, molecular targeted therapy for gastric cancer has received increasing attention. Human epidermal growth factor receptor 2 (HER2) is a receptor tyrosine kinase that belongs to the human epidermal growth factor receptor family (EGFR). The HER2 gene is located in the 17q21 region of the human chromosome, and it is responsible for encoding a transmembrane protein that is 185 kDa in molecular weight. The process of dimerisation induces the phosphorylation of tyrosine residues within the intracellular protein kinase domain, thereby initiating the downstream mitogen-activated protein kinase Ras/Raf/MEK/ERK (MAPK) pathways and phosphatidylinositol 3-kinase/protein kinase B/mammalian target of rapamycin (PI3K/Akt/mTOR) pathways. This subsequently causes abnormalities in the division, proliferation, differentiation, and anti-apoptotic signals of GC cells, promoting the invasive growth of GC [2, 3] (Fig. 1).

HER2 is an important target and biomarker for the treatment of advanced gastric cancer, which is characterised by high malignancy and poor clinical prognosis, with a 5-year survival rate of only 5% to 20% [4]. It has been reported that between 15% and 20% of patients diagnosed with gastric cancer exhibit HER2 protein overexpression or gene amplification [5–7]. These observations are of crucial significance in understanding the aetiology of the disease. It is noteworthy that the expression of HER2 is closely related to the Lauren classification of gastric cancer, with a significantly higher HER2 positivity rate in intestinal-type gastric cancer compared to diffuse-type. Moreover, HER2 amplification has been shown to be positively correlated with tumour microvascular density (MVD) and has been identified as an independent factor affecting the prognosis of patients with gastric cancer [8, 9].

HER2 status testing is performed on biopsy tissue samples using immunohistochemistry (IHC) and fluorescence in situ hybridization (FISH) or in situ hybridization (ISH) [10]. According to the guidelines jointly issued by the College of American Pathologists, the American Society for Clinical Pathology, and the American Society of Clinical Oncology, an IHC score of 0 or 1+ is defined as HER2‑negative, a score of 2+ is considered equivocal, and a score of 3+ is HER2‑positive. Patients with an IHC score of 2+ should undergo further testing by FISH or ISH. Based on FISH/ISH results, tumors are classified as HER2‑positive or HER2‑negative. Specifically, IHC 3+ or IHC 2+ with a positive FISH/ISH result is defined as HER2‑positive (or HER2‑overexpressing), whereas IHC 1+ or IHC 2+ with a negative FISH/ISH result is classified as having HER2‑low expression. The latest clinical guidelines from the Chinese Society of Clinical Oncology (CSCO) further refine this classification based on the combination of HER2 protein expression (by IHC) and HER2 gene copy number (by FISH/ISH), dividing HER2 status into four categories: HER2‑high (IHC 3+ or IHC 2+/FISH+), HER2‑medium (IHC 2+/FISH‑), HER2‑low (IHC 1+, irrespective of FISH status), and HER2‑null (IHC 0, irrespective of FISH status). This new classification moves beyond the traditional dichotomy of HER2‑positive versus HER2‑negative and is increasingly driven by clinical needs. HER2‑low and HER2‑ultralow breast cancer (BC) have recently been proposed as new subcategories of HER2 BC. HER2‑low expression is defined as IHC 1+ or IHC 2+ in the absence of FISH amplification (i.e., FISH‑), whereas HER2‑ultralow expression is characterized by ≤ 10% of invasive cancer cells showing faint or incomplete membrane staining that does not meet the criteria for IHC 1+ [11, 12]. In the context of gastric cancer, the subclassification of HER2‑ultralow expression is currently under investigation. This refined classification of HER2 has profound clinical significance, manifested primarily through directing novel targeted therapies, elucidating prognostic differences, and facilitating a reconceptualization of disease biology.

With advancements in tumor molecular biology, the field of oncology has witnessed the widespread adoption of targeted, anti‑angiogenic, and immunotherapies, as well as combination regimens, in the treatment of advanced gastric cancer. At present, standard anti-HER2 targeted therapies such as trastuzumab combined with chemotherapy have become the first-line treatment for HER2-positive advanced gastric cancer, however, no therapeutic efficacy was observed in patients with HER2-low expressing tumors [13, 14]. Nevertheless, the high degree of tumour heterogeneity in gastric cancer, the poor prognosis for patients, and the high rate of drug resistance remain significant obstacles to its utilisation. The mechanisms of resistance to trastuzumab treatment have been shown to be related not only to the status of HER2 itself (including abnormal expression of HER2, HER ligands, and regulation of downstream signalling pathways) [15–17], but also to the abnormal activation of classic signalling pathways such as PI3K/AKT and MEK/ERK [18, 19] and non-classical signalling pathways related to mitosis [20]. As indicated by the extant literature, fluctuations in metabolism and immune regulation within the tumour microenvironment of patients diagnosed with gastric cancer have the potential to engender resistance to trastuzumab [21–23]. Furthermore, for patients with unresectable, locally advanced, or metastatic HER2‑negative gastric cancer, studies have demonstrated that nivolumab plus chemotherapy yields a median overall survival (OS) of 14.4 months in the subgroup with a PD‑L1 combined positive score (CPS) ≥ 5 [24]. However, among patients with CPS < 5, particularly those with CPS < 1, the vast majority appear unlikely to derive benefit from immunotherapy. Notably, in real-world clinical practice, patients with CPS < 5 constitute more than 50% of the population [25]. Consequently, a substantial subset of patients remain unlikely to benefit from the aforementioned treatment regimens.

Antibody-drug conjugates (ADCs) have been introduced into clinical practice as a novel precision therapy for tumors and have gradually demonstrated significant potential. The combination of the targeting ability of antibodies with highly potent cytotoxic agents, through a “biological missile” design, significantly improves the therapeutic index [26, 27]. By the end of 2025, a minimum of 19 ADCs will have been approved and marketed on a global scale, achieving noteworthy efficacy in treating various malignancies, including lymphoma, breast cancer, lung cancer, multiple myeloma, head and neck cancer, cervical cancer, and urothelial carcinoma [28–37]. Furthermore, it should be noted that a number of ADCs are currently undergoing clinical research in various stages. Furthermore, significant strides have been made in the field of ADCs therapies for the treatment of HER2-positive advanced gastric cancer, both as a subsequent line of therapy and as a primary treatment. For instance, in the DESTINY-Gastric01 phase III clinical trial [38], T-DXd exhibited a considerably higher objective response rate and overall survival in comparison to conventional chemotherapy in patients with HER2-positive advanced gastric cancer, thereby leading to a fundamental shift in clinical practice, and the introduction of this drug also signifies that extending the benefits of anti‑HER2 therapy to patients with HER2-low tumors has become possible. Moreover, the combination of immune checkpoint inhibitors with anti-HER2 therapy has also shown significant potential. The results of the KEYNOTE-811 study indicated that the combination of the anti-PD-1 antibody, pembrolizumab, with trastuzumab and chemotherapy as a first-line treatment significantly improves the objective response rate in patients with HER2-positive advanced gastric cancer [39]. Nevertheless, the distinctive toxic reactions of ADCs, including interstitial lung disease/pneumonitis, and the inevitable issue of drug resistance, persist as significant clinical concerns [26, 40]. The present article will summarise the clinical application of ADC in HER2-positive and HER2-low advanced gastric cancer, focusing on the following areas: the drug mechanisms of action, clinical efficacy and safety research and management, mechanisms of resistance, and countermeasures. The article will provide valuable insights into the current treatment status and future prospects of ADCs.

### Structure and mechanism of action of ADCs 

**Fig. 2:**
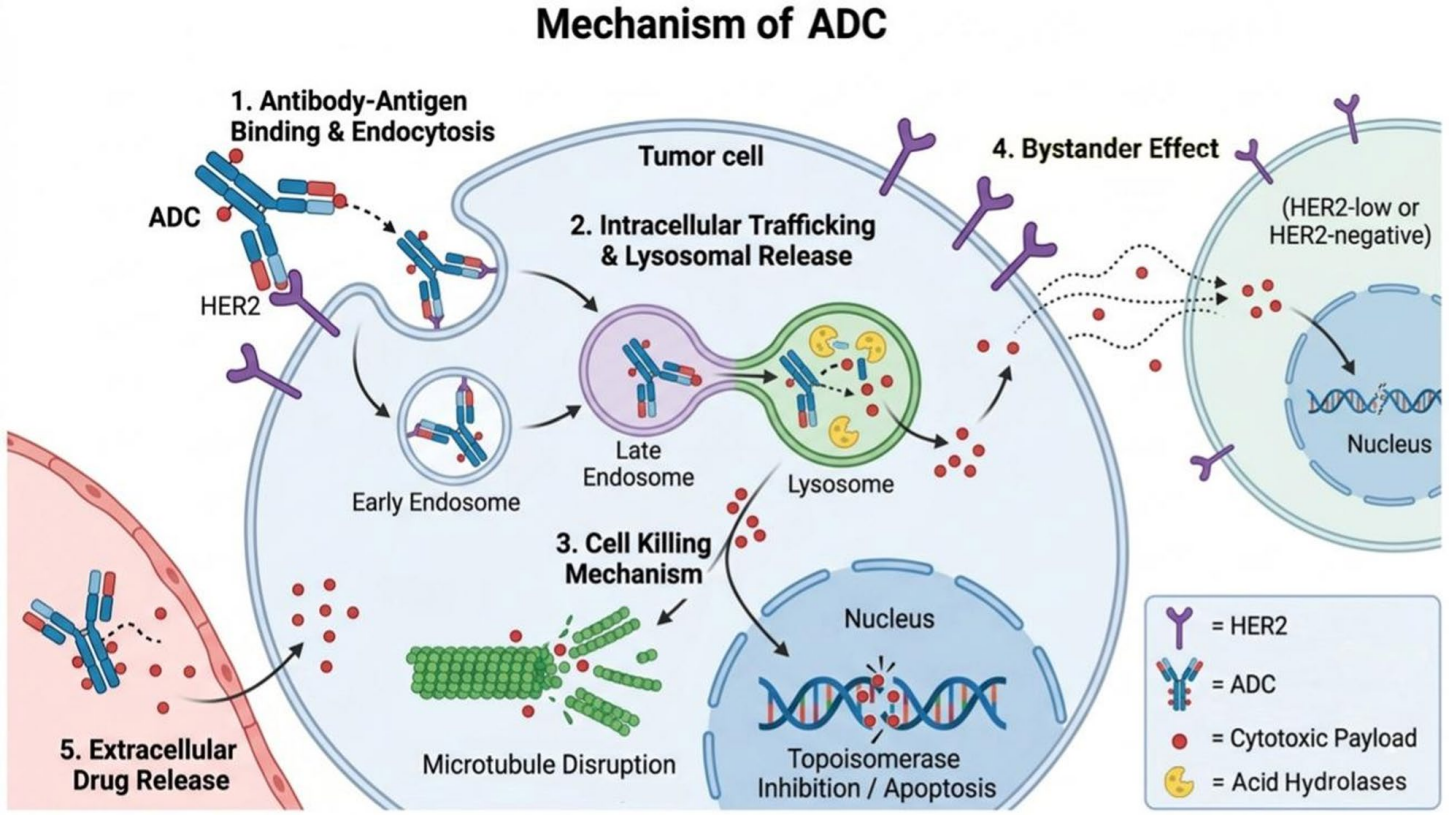
The working mechanism of ADC therapeutics. Antibody-drug conjugates utilize monoclonal antibodies to specifically bind to tumor cell surface antigens. Following internalization, the cytotoxic payload (e.g., microtubule inhibitors or DNA-damaging agents) is released in lysosomes, selectively killing tumor cells. Additionally, the “bystander effect” enables targeting of adjacent antigen-negative cells, achieving potent antitumor efficacy with reduced systemic toxicity

ADCs are a class of molecularly targeted anti-tumor biologics, with their mechanism of action primarily dependent on unique structural design and specific interactions with HER2-positive cells. The composition of an ADC is typically as follows: antibodies that specifically recognize tumor cells; highly active cytotoxic payloads (small molecule drugs); and a chemical linker that connects the antibody and payload [41]. The core mechanism is initiated by the antibody component, which specifically recognises and binds to overexpressed HER2 receptors on the surface of tumor cells. This binding event has been shown to trigger receptor-mediated endocytosis, thereby facilitating the internalization of the ADC-antigen complex into the cell [42]. Subsequently, the complex is transported to the lysosome, where the linker is cleaved or degraded in the acidic lysosomal environment and by specific enzymes (e.g., cathepsin B), thereby releasing the highly active cytotoxic drug [43]. The released payload, which may comprise microtubule inhibitors or topoisomerase inhibitors, acts on key intracellular targets, inducing DNA damage or inhibiting microtubule function. Ultimately, this triggers the apoptosis of tumour cells [44]. Furthermore, ADCs have been observed to exert cytotoxic effects on HER2-negative tumour cells via the bystander effect. When HER2-positive cells are bound by ADCs, elimination of the bound cells results in the release of an active payload. This diffuses to adjacent HER2-negative tumour cells, exerting a cytotoxic effect on these cells [45]. The combination of the high specificity of monoclonal antibodies (mAbs) with the cytotoxic potency of small molecule drugs results in ADCs offering more precise tumour-killing characteristics compared to conventional chemotherapy agents. This precision selectively reduces adverse reactions caused by off-target effects, resulting in improved safety and tolerability, and enables accurate and efficient elimination of cancer cells. The mechanism of action of the ADC is illustrated in Fig. 2.

### Clinical Efficacy and Safety of ADCs in HER2-Positive or HER2-low advanced Gastric Cancer

#### Trastuzumab emtansine (first-generation ADC)

Trastuzumab emtansine (T-DM1) is the first-generation antibody-drug conjugate formed by linking trastuzumab and the microtubule inhibitor derivative DM1 through a thioether bond, with a DAR of 3.5. The delivery of DM1 to HER2-overexpressing cells is facilitated via receptor-mediated endocytosis. The drug’s mechanism of action involves binding to microtubule proteins, thereby preventing microtubule polymerization and formation. This, in turn, results in cell cycle arrest in the metaphase of mitosis, leading to the demise of tumour cells. This pharmaceutical agent has yielded notable outcomes in the management of HER2-positive breast cancer, and has been sanctioned as the inaugural ADC to be endorsed on a global scale for monotherapy in the context of solid tumours. A number of studies have been conducted on the use of T-DM1 in the treatment of HER2-positive gastric cancer. However, the pivotal phase II/III GATSBY study failed to achieve the primary endpoint of enhancing overall survival, thereby suggesting that T-DM1 may not be appropriate for the distinctive tumour microenvironment of gastric cancer, thus limiting its efficacy [46].

Another GATSBY study [47] focused on T-DM1 in patients with HER2-positive locally advanced or metastatic gastric or gastroesophageal junction adenocarcinoma from the Japanese population, enrolling 82 patients, of whom 48 received T-DM1 treatment (2.4 mg/kg weekly). The results showed that the median overall survival for the T-DM1 and paclitaxel treatment groups was 11.8 months and 10 months, respectively. In comparison with paclitaxel, T-DM1 at a dose of 2.4 mg/kg per week resulted in a lower incidence of grade 3 or higher adverse events (AEs) (52.1% vs. 68.2%) and serious AEs (14.6% vs. 18.2%). During the course of treatment, no fatal AEs were observed. The study concluded that T-DM1 did not prolong overall survival in comparison with paclitaxel. Consequently, the efficacy of T-DM1 in HER2-positive advanced gastric cancer patients is subject to significant limitations, which may be attributable to the poor membrane permeability of DM1 and the low “bystander” effect of the drug due to the non-cleavable linker of T-DM1. Meanwhile, the different levels of HER2 expression in primary and metastatic gastric cancer lesions are closely related to the poor efficacy of T-DM1. Moreover, the most common adverse reactions associated with T-DM1 are thrombocytopenia and hepatotoxicity. As indicated by reports from international sources, the incidence of thrombocytopenia of all grades in patients treated with T-DM1 ranges from approximately 25% to 31%, with a grade ≥ 3 incidence ranging from about 2% to 15%. However, the risk of thrombocytopenia is higher in Asian populations, with an incidence of all grades reaching 52.5% to 69.8%, and a grade ≥ 3 incidence of about 29.8% to 45% [48]. The precise mechanism underlying this adverse reaction remains to be elucidated; however, it may be associated with the endocytosis of T-DM1 by megakaryocytes. In summary, following the failure of treatment in cases of HER2-positive advanced gastric cancer, a substantial number of clinical studies are required to substantiate the efficacy and safety enhancement of alternative treatment regimens.

#### Trastuzumab deruxtecan (second generation)

Trastuzumab deruxtecan (T-DXd or DS-8201a): This pharmaceutical compound is a second-generation ADC, which employs a cleavable tetrapeptide linker to connect a humanised anti-HER2 IgG1 antibody with the topoisomerase I inhibitor DXd [26]. The effective payload potency of this drug is ten times greater than that of the active metabolite of the topoisomerase I inhibitor irinotecan [7]. The DAR of this pharmaceutical compound is approximately 8, indicating that the number of cytotoxic drugs per antibody molecule is considerably augmented, consequently amplifying its cytotoxic potency [49]. It is noteworthy that the DXd released by T-DXd is membrane-permeable, thereby enabling it to diffuse out of internalised tumour cells and kill adjacent tumour cells with low or even negative HER2 expression. The bystander effect is a crucial factor in addressing the common problem of tumour heterogeneity in gastric cancer [45]. Research has indicated considerable spatiotemporal heterogeneity in HER2 expression within gastric cancer tissues, a phenomenon that poses challenges for conventional targeted therapies. However, the utilisation of ADCs exhibiting a “bystander effect” has been demonstrated to be a more efficacious approach in addressing this heterogeneity [50]. In patient‑derived xenograft (PDX) models, T‑DXd significantly inhibited the growth of both HER2‑low tumor cells and HER2‑positive tumor cells resistant to trastuzumab or ado‑trastuzumab emtansine (T‑DM1) [51]. These findings indicate that T‑DXd exhibits potent activity and marked antitumor efficacy against HER2‑low‑expressing cells [51].

The DESTINY-Gastric01 study (NCT03329690, Study Registration Dates: 2017-10-30) [38] is a randomised, open-label phase II trial. The target population for this study consists of adult patients diagnosed with locally advanced or metastatic HER2-positive gastric or gastroesophageal junction adenocarcinoma who have received a minimum of two treatment regimens, including trastuzumab. Among the 187 treated patients, 125 received T-DXd (6.4 mg/kg every 3 weeks) and 62 received chemotherapy, of whom 55 received irinotecan and 7 received paclitaxel. The findings indicated that the median overall survival (OS) in the T-DXd group exhibited a significantly longer duration when compared to the chemotherapy group (irinotecan or paclitaxel) (12.5 months vs. 8.4 months). The objective response rate (ORR) exhibited a substantial enhancement (51.3% vs. 14.3%). Concurrently, T-DXd exhibited superior outcomes in comparison to the chemotherapy group with respect to progression-free survival (PFS), disease control rate (DCR), and duration of response (DOR). In comparison with the chemotherapy group, T-DXd demonstrated a substantial enhancement in the DCR to 85.7%, and the DOR to 11.3 months. This finding suggests that a significant proportion of patients diagnosed with later-line HER2-positive advanced gastric cancer can achieve disease control following T-DXd treatment, with a median DOR that approaches one year. Although T-DXd demonstrated significant survival benefits, concerns regarding safety must also be addressed. The study found that, in comparison with the chemotherapy group, the T-DXd group exhibited a higher incidence of grade 3 or higher adverse reactions, including a decrease in neutrophil count (51% vs. 24%), anaemia (38% vs. 23%), and a reduction in leukocyte count (21% vs. 11%). Approximately 10% of patients exhibited drug-related interstitial lung disease or pneumonia, with a single instance of drug-related mortality observed in the T-DXd group. Moreover, the efficacy and safety data for T-DXd from the exploratory HER2-low expression cohorts were subsequently reported [52]. In Cohort 1 (IHC 2+/ISH−), the ORR was 26.3%, while median DOR, OS, and PFS were 7.6, 7.8, and 4.4 months, respectively. In Cohort 2 (IHC 1+), the ORR was 9.5%, with median DOR, OS, and PFS of 12.5, 8.5, and 2.8 months, respectively. Regarding safety, in cohorts 1 and 2, the most frequent grade ≥ 3 treatment-emergent adverse events (TEAEs) were anemia (30.0% and 29.2%), neutrophil count decreased (25.0% and 29.2%), and decreased appetite (20.0% and 20.8%). Therefore, T-DXd demonstrates therapeutic efficacy in patients with both HER2‑positive and HER2‑low advanced gastric cancer, thereby providing a novel therapeutic alternative for this population that has historically lacked effective targeted therapy.

Following the development of breakthroughs in ADCs for later-line treatments, the therapeutic landscape is evolving gradually, with the aim of exploring the potential of these drugs in first-line and second-line treatments for HER2-positive advanced gastric cancer. The DESTINY-Gastric02 study (NCT04014075) further validated the efficacy and safety of T-DXd in the second-line treatment of HER2-positive advanced gastric cancer in Western populations, providing new treatment options for patients who have progressed after previous treatment with trastuzumab-containing regimens [53, 54]. The present study also provides substantial clinical evidence for third-line and subsequent treatments of HER2-positive gastric cancer. As of April 2021, the study included 79 patients. Following treatment with T-DXd (6.4 mg/kg intravenously every 3 weeks), a complete disappearance of tumors was observed in four patients, while significant tumor shrinkage was noted in 29 patients. Following a median follow-up period of 10.2 months, the ORR was recorded as 41.8% (95% CI, 30.8–53.4), and the DOR was determined to be 8.1 months (95% CI, 5.9-NE). The PFS was found to be 5.6 months (95% CI, 4.2–8.3), and the OS was 12.1 months (95% CI, 9.4–15.4). The safety profile of the drug was consistent with the previously reported safety characteristics of DS-8201a. The most common treatment-emergent adverse events (TEAEs) that were reported as grade ≥ 3 included anaemia (13.9%), nausea (7.6%), neutropenia (7.6%), and leukopenia (6.3%). Serious drug-related TEAEs occurred in 10 patients (12.7%), and drug-related TEAEs associated with death occurred in 2 patients (2.5%), primarily due to interstitial lung disease/pneumonia. A comparison of the second-line treatment of ramucirumab plus paclitaxel in the RAINBOW trial with that of T-DXd in the DESTINY-Gastric02 trial reveals higher response rates and survival outcomes in the latter. At present, there are several key phase III studies underway that are aiming to advance ADC treatment and which are expected to effect a change in the existing treatment landscape. The DESTINY-Gastric03 (NCT04379596) study explores combination regimens of T-DXd with cytotoxic chemotherapy or immune checkpoint inhibitors, while the DESTINY-Gastric04 (NCT04704934) study directly compares the efficacy and safety of T-DXd versus the standard second-line chemotherapy of ramucirumab plus paclitaxel (RAM+PTX) for the second-line treatment of HER2-positive unresectable or metastatic gastric cancer/gastroesophageal junction adenocarcinoma (GC/GEJA) [55]. Recent data from this study demonstrate compelling dual positive results in terms of OS and PFS, thus confirming the significant superiority of T-DXd in the second-line treatment of HER2-positive gastric cancer. The study found OS was significantly improved in the T‑DXd group compared with the ramucirumab-paclitaxel group (median survival, 14.7 months vs. 11.4 months; hazard ratio for death, 0.70). Significant benefits were also demonstrated in PFS (hazard ratio for disease progression or death, 0.74) and in confirmed objective response (44.3% in the trastuzumab deruxtecan group vs. 29.1% in the ramucirumab-paclitaxel group) [55]. The basis of these studies is the potential synergistic effects demonstrated by T-DXd in preclinical models when combined with immunotherapy or other targeted drugs [56].

The DESTINY-Gastric06 study [57] is a bridging study conducted in China based on DESTINY-Gastric01 [38]. The present study constitutes an open-label, single-arm, multi-centre, phase II clinical trial that enrolled Chinese patients diagnosed with HER2-positive (IHC3+orIHC2+) advanced gastric cancer who had previously received a minimum of two treatment regimens (including fluorouracil and platinum-based drugs). Patients received T-DXd 6.4 mg/kg intravenous infusion every 3 weeks.The study results demonstrated that, among 73 patients diagnosed with HER2-positive gastric cancer, at a median follow-up period of 10.2 months, the mPFS was 5.7 months, and the mOS was 11.1 months. Sensitivity analysis revealed that, following the censorship of four deaths due to COVID-19, the mOS was extended to 12.4 months. Moreover, with regard to safety, decreased white blood cell count was the most common Grade 1–2 adverse event, with an incidence of 53.7% (51/95). The occurrence of any-grade interstitial lung disease (ILD) was only 3.2%. These data are consistent with the results of the DESTINY-Gastric01 study, thus further validating the value of T-DXd in the treatment of HER2-positive advanced gastric cancer.

In conclusion, T-DXd provides a unique mechanistic advantage in overcoming HER2 heterogeneity in gastric cancer through its high drug-to-antibody ratio and bystander effect. The DESTINY-Gastric series of studies has established its pivotal role in the treatment of HER2-positive advanced gastric cancer, from later-line to second-line therapy. In comparison with conventional chemotherapy, T-DXd has been demonstrated to significantly enhance the objective response rate and overall survival. Nevertheless, its clinical application demands meticulous consideration of safety concerns. Myelosuppression is the most common severe adverse event in clinical practice, while interstitial lung disease, despite its relatively low incidence, has emerged as a key focus in clinical medication management due to its potential lethality [55, 58]. T-DXd has led to a substantial enhancement in the therapeutic landscape for HER2-positive advanced gastric cancer, and T-DXd has broadened the population that can benefit from anti-HER2 therapy to include those with HER2-low advanced gastric cancer. Nevertheless, further exploration of its use in first-line treatment and combination strategies is required, with the objective of identifying the optimal balance between efficacy gains and toxicity control.

#### Disitamab vedotin (third generation)

Disitamab vedotin (RC48) is a novel antibody-drug conjugate consisting of a humanised anti-HER-2 monoclonal antibody targeting the extracellular domain, a valine-citrulline (VC) linker, and the cytotoxic agent monomethyl auristatin E (MMAE) [59]. As a third-generation HER-2-ADC, it exhibits a superior antibody affinity for HER-2 antigens and elevated levels of cellular toxicity in comparison to T-DXd. The antibody component of the drug inhibits the downstream signalling pathways activated by HER-2, thereby exerting a synergistic effect on the killing of tumour cells in conjunction with the cytotoxic drug. Furthermore, it has been demonstrated to interfere with cancer cell transcription, growth, proliferation, and division, thus exhibiting significant antitumour efficacy. 

A phase I trial of RC48 demonstrated favorable outcomes in patients with HER2-positive advanced GC [60], a total of 57 patients were enrolled, and 2.5 mg/kg Q2W was determined as the recommended phase II dose (RP2D). Overall, the ORR and DCR were 21.0% (12/57) and 49.1% (28/57), respectively. Notably, responses in patients with HER2 IHC2+/FISH− were similar to those in patients with IHC2+/FISH+ and IHC3+, with ORRs of 35.7% (5/14), 20.0% (2/10), and 13.6% (3/22), respectively. In patients pretreated with HER2‑targeted agents, RC48 also demonstrated favorable efficacy, with an ORR of 15.0% (3/20) and a DCR of 45.0% (9/20). RC48 was well tolerated, and the most common grade ≥ 3 treatment‑related adverse events (TRAEs) included neutropenia (19.3%), leukopenia (17.5%), hypoesthesia (14.0%), and increased conjugated bilirubin (8.8%). A total of four deaths occurred during the study period, of which three were considered related to RC48. It has demonstrated promising antitumor activity in the HER2‑positive solid tumors, particularly gastric cancer with HER2 IHC 2+/FISH− status.

The RC48-C008 study [61] constitutes a single-arm, multicentre phase II clinical trial led by Chinese investigators with the objective of treating HER2-overexpressing (immunohistochemistry IHC2+or3+) advanced gastric cancer. The study included a total of 125 patients with locally advanced or metastatic gastric cancer who had progressed after at least second-line treatment, and were treated with RC48 monotherapy at a dose of 2.5 mg/kg every 2 weeks. The results demonstrated that the DCR of RC48 in patients with second-line or higher was 42.4%, the ORR was 24.8%, the median progression-free survival (mPFS) was 4.1 months, and the median overall survival (mOS) was 7.9 months. Adverse events included a decrease in white blood cells, fatigue, hair loss, a decrease in blood platelets, anaemia, and increased levels of aspartate aminotransferase. Severe adverse events (SAEs) occurred in 45 patients, with the primary SAE associated with RC48 being leukopenia.

Collectively, RC48 monotherapy demonstrated a tolerable safety profile and promising antitumor activity against gastric cancer and other solid tumors expressing HER2. Based on these results, RC48 has been approved in China for the third-line treatment of HER2-overexpressing gastric cancer at the recommended dose of 2.5 mg/kg once every 2 weeks.

Nevertheless, challenges remain, as only a subset of patients can benefit from RC48 monotherapy. Studies have indicated that ADCs induce tumor‑specific adaptive immunity and promote T‑cell infiltration into the tumor microenvironment, whereas ICIs reinvigorate exhausted T cells and enhance antitumor immune responses [62]. Preclinical studies have demonstrated that ADCs upregulate PD‑L1 and major histocompatibility complex class I (MHC‑I) on tumor cells, and that the combination of these agents with immunotherapy yields synergistic antitumor activity [63]. This synergistic effect not only improves the overall treatment efficacy, but may also help overcome resistance associated with monotherapy (MT). Several studies have indicated that HER2-targeted ADCs combined with PD-1 inhibitors exert synergistic efficacy in the treatment of HER2-expressing gastric cancer.

A multicentre real-world study [64] was conducted to compare the efficacy of ICIs combination with RC48 monotherapy as third-line treatment for patients with HER2-positive or HER2-low advanced or metastatic gastric cancer. The study found that the combination therapy was superior in terms of treatment efficacy, with manageable safety. The study enrolled 45 patients. In the present study, 25 patients received a combination of RC48 and ICIs (RC48 was administered at the same dosage used in RC48 monotherapy; tislelizumab was administered intravenously at a dosage of 200 mg once every three weeks), while 20 patients received RC48 alone (RC48 was administered intravenously at a dosage of 2.5 mg/kg every two weeks). In comparison with RC48, patients receiving the combination of RC48 and ICIs exhibited a superior ORR of 36.0% and a DCR of 80.0%. mPFS was superior to that of the RC48 group (6.2 months vs. 3.9 months). The most prevalent adverse events included leukopenia, neutropenia, fatigue, sensory reduction, and alopecia. Furthermore, Grade 3–4 adverse events were observed in 7 cases (35.0%) in the RC48 group and 10 cases (40.0%) in the RC48 plus ICIs group.

Another report (RC48-C013) [65] described an open-label, multicentre, phase I clinical trial that enrolled 56 patients, including 30 GC/GEJC patients and 26 patients with other solid tumors, with HER2 IHC ≥ 1 or in situ hybridisation (ISH) positive, and who were ineffective against at least one treatment method, or these patients could not tolerate or access standard treatment. The dosage of RC48 was 2.5 mg/kg in combination with Toripalimab 3 mg/kg, administered every two weeks (RP2D). In patients with GC/GEJC cancer treated at the RP2D (*n* = 24), the ORR was 50% (11/22, 95% CI 28%, 72%), the median PFS was 5.1 months (95% CI 1.4, 7.3), and the median OS was 14.0 months (95% CI 6.3, NE). Among the RP2D-treated GC/GEJC cancer patients, clinical benefit was observed in both HER2‑positive and HER2‑low populations, with ORRs of 56% (5/9, 95% CI 21%, 86%) and 46% (6/13, 95% CI 19%, 75%), median PFS values of 7.8 months (95% CI 0.9, NE) and 5.1 months (95% CI 1.2, 6.9), and median OS values of NE (95% CI 4.3, NE) and 14.0 months (95% CI 5.1, NE), respectively. The combination of the two drugs was found to be safe and manageable, with encouraging signs of efficacy.

A separate multicenter retrospective study [66] enrolled 34 patients in the RC48 plus anti‑PD‑1immunotherapy (IT) group and 34 in the RC48 monotherapy (MT) group. All patients received RC48 at 2.5 mg/kg every 2 weeks. PD-1 inhibitors were administered according to the recommended instructions. The PFS was significantly longer in the combination-therapy (CT) group compared with the MT group (5.3 vs. 3.8 months). Similarly, The OS was significantly improved in the CT group (10.0 vs. 6.8 months). The ORR and DCR were higher in the CT group (41.18% vs. 14.71%). Furthermore, subgroup analyses revealed that patients in the CT group experienced longer PFS and OS, particularly those with high HER2 expression or a PD‑L1 combined positive score (CPS) of ≥ 1. The combination therapy demonstrated an acceptable tolerability profile and manageable adverse events. Moreover, the most common grade 3‑5 TRAEs included decreased WBC count, decreased neutrophil count, and anemia. No new safety risks were observed.

Several real-world cohort studies and clinical trials have repeatedly shown that RC48 has distinct antitumor activity in advanced gastric cancer, with a ‘continuous spectrum’ of efficacy across HER2 medium (IHC 2+/FISH⁻) and HER2 low (IHC 1+) tumors [59, 67]. This phenomenon is largely explained by the bystander effect of RC48, in which the released cytotoxic payload exerts cytotoxic effects on both antigen-positive cells and adjacent HER2-low or heterogeneous cells, thereby overcoming the inherent limitations of conventional monoclonal antibody therapies that are restricted by target expression thresholds [68]. RC48, given its activity against HER2‑low/heterogeneous tumors and its high response rates in combination regimens, offers a valuable complementary option for patients whose HER2 expression is below the conventional ‘positive’ cutoff but who may derive benefit from ADC‑based treatment.

### Other novel ADCs

#### ARX788

ARX788 represents a next-generation, site-specific anti-HER2 ADC that employs a unique unnatural amino acid conjugation technology to link Trastuzumab with a potent microtubule inhibitor, Amberstatin (AS269). The DAR of the latter is 1.9. Compared to T‑DM1 across a panel of cancer cell lines in vitro, ARX788 demonstrated superior potency in cell lines with HER2-low expression, yet displayed no toxicity toward normal cardiomyocytes. The pharmaceutical compound has been demonstrated to inhibit tumor growth to a significant degree, and has been shown to demonstrate superior efficacy in comparison to T-DM1 in both HER2-high and HER2-low expression xenograft models [69]. Moreover, the xenograft studies conducted in patients diagnosed with breast cancer and gastric cancer substantiate the robust antitumour activity of ARX788 in HER2-positive and HER2-low expression tumors, in addition to T-DM1-resistant models [69].

A multicentre, dose-escalation phase I clinical trial has been initiated [70], with 30 patients enrolled; 27 (90%) of whom had previously received Trastuzumab monotherapy, and 12 (40%) had received second-line or higher treatment. Of the subjects who received ARX788 treatment, 9 patients were administered a dose of 1.3 mg/kg, 14 patients received a dose of 1.5 mg/kg, and 7 patients received a dose of 1.7 mg/kg. The study results demonstrated that ARX788 exhibited optimal anti-tumour efficacy, with the ORR of 37.9%, the DCR of 55.2%, and the DOR of 8.4 months. The mPFS and mOS were 4.1 months and 10.7 months, respectively. The efficacy of ARX788 is comparable to the results published for T-DXd, yet it is superior to those of the RC48 study. The study found that ARX788 was well tolerated by patients with HER2-positive advanced gastric adenocarcinoma. The majority of adverse events observed were mild or moderate, with only four patients experiencing grade 3 adverse events related to ARX788. No grade 4 or 5 adverse events were observed. Patients who experienced grade 3 toxicity all achieved relief or recovery after treatment, indicating that the safety is controllable.

On 18 March 2021, ARX788 was designated as an orphan drug by the FDA for the treatment of HER2-positive gastric cancer and gastroesophageal junction cancer. Presently, the international multicentre, randomised, open-label, positive-controlled phase II/III ACE-Gastric-02 study (CTR20211583) of ARX788 monotherapy for second-line treatment of HER2-positive gastric cancer or gastroesophageal junction cancer is being conducted globally.

#### SYD985

SYD985 (Trastuzumab duocarmazine) is a second-generation HER2-targeted novel ADC. The pharmaceutical compound under discussion consists of trastuzumab linked to a duocarmycin analogue through a cleavable valine-citrulline linker. The DAR has been calculated to be 2.8. The antibody component of the ADC targets and binds to HER2 on cancer cells, leading to the internalization of the ADC. It has been demonstrated that the payload becomes inactive once it has been attached to the antibody. Following the process of linker hydrolysis and cleavage, the cytotoxic drug duocarmycin is reactivated. Subsequently, it binds to the guanine residue in the minor groove of DNA, resulting in alkylated DNA. This process initiates DNA damage, which ultimately leads to the death of tumor cells [71]. Furthermore, duocarmycin demonstrates high permeability to membranes and effectively induces the “bystander” effect, resulting in the killing of non-target tumor cells.

To assess the efficacy of SYD985 against T‑DM1‑resistant tumors, multiple patient‑derived models of T‑DM1 resistance were established. Characterization of these models revealed that previously described resistance mechanisms—including HER2 down regulation, impaired lysosomal function, and upregulation of drug efflux pumps—accounted for the observed resistance, suggesting that the mechanisms of T‑DM1 resistance are restricted and largely elucidated. Importantly, SYD985 demonstrated significant efficacy in these models, indicating that improved ADC design can overcome resistance to first‑generation ADCs [72].

Banerji et al. [73] conducted a phase I dose-escalation study [NCT02277717] based on previous research, enrolling 39 patients to assess the safety of SYD985. Subsequent to this, a phase I dose-expansion study was conducted, encompassing 146 patients, with the objective of evaluating the efficacy of the drug in patients diagnosed with metastatic solid tumours and HER2-expressing breast cancer. In the cohort of 16 patients with gastric cancer that was escalated in dosage, a single patient (95% CI: 0.2–30.2) demonstrated a partial response (PR), resulting in a mPFS of 3.2 months (95% CI: 1.6–5.3). The phase I clinical study provided further confirmation of the anti-tumor activity of SYD985. The most prevalent treatment-related adverse events (grades 1–4) included fatigue (33%), conjunctivitis (31%), and dry eye (31%). In the present study, 104 patients (71%) experienced at least one ocular adverse event, with 10 patients (7%) reporting grade 3 events.

In August 2022, the US FDA granted orphan drug designation to SYD985 for the treatment of gastric cancer and gastroesophageal junction adenocarcinoma. A multicentre, open-label, single-arm phase I/Ib study (NCT04602117) is currently ongoing to determine the effectiveness of SYD985 in combination with paclitaxel for treating advanced gastric cancer.

#### MRG002

MRG002 is a novel ADC that incorporates a sugar-modified trastuzumab, which exhibits a strong binding affinity for HER2, and the potent microtubule inhibitor MMAE. These components are interconnected by an enzymatically cleavable valine-citrulline (Vc) linker. The sugar-modified trastuzumab demonstrates a stronger affinity for the HER2 protein, exhibiting a DAR of 3.8 [74]. MRG002 has been demonstrated to circumvent certain mechanisms of drug resistance, including defects in the PTEN-PI3K/AKT pathway, autocrine production of EGF-related ligands, and impairment of ADCC activity. This process is known as HER2-mediated endocytosis, which results in the lysosomal release of the cytotoxic agent MMAE from the internalised ADC [75]. In contrast to trastuzumab, which primarily depends on ADCC activity and immune responses for cancer cell elimination, MRG002 effectively eliminates cancer cells directly through the use of potent cytotoxins. Furthermore, MRG002 is not influenced by the immune status. Its high level of fucosylation, which has been demonstrated to reduce ADCC activity, may help to reduce side effects in clinical settings [76]. Preclinical studies had previously demonstrated the efficacy of MRG002 in the inhibition of tumors in both HER2-overexpressing breast and gastric cancer models, both in vitro and in vivo. MRG002 demonstrated a superior therapeutic efficacy profile in comparison to TDM-1 in models of gastric cancer. The drug also demonstrated notable anti-tumor activity in models of gastric cancer that were resistant to trastuzumab and TDM-1. Moreover, the combination of MRG002 and an anti-PD-1 antibody exhibited enhanced anti-tumor activity in comparison with MRG002 utilised in isolation [74]. This combination therapy was identified as a potentially efficacious treatment option for patients who had both PD-1 resistance and HER2 overexpression. A phase I study (NCT04941339) is currently underway to evaluate the efficacy of MRG002 as a monotherapy in patients diagnosed with HER2-positive recurrent or refractory gastric cancer [77].

Additionally, clinical trials in phases I and II (NCT04492488) are examining MRG002 in patients with HER2-positive advanced solid tumors, such as locally advanced or metastatic gastric cancer and gastroesophageal junction cancer [78]. Concurrently, a phase II clinical study (NCT05141747) was assessing MRG002 for the treatment of HER2-positive or low HER2-expressing locally advanced or metastatic GC/GEJC [79]. Collectively, these clinical studies aim to introduce new treatment options for patients diagnosed with HER2-positive gastric cancer.

#### SHR-A1811

SHR-A1811 is a third-generation ADC drug that comprises trastuzumab, a cleavable tetrapeptide linker (GGFG), and a novel topoisomerase I inhibitor payload (SHR169265), with a DAR of 6.0 [80]. This ADC has been engineered to target HER2 on the surfaces of tumour cells. The release of toxins is catalysed by cathepsin-mediated hydrolysis, resulting in the inhibition of DNA topoisomerase I and the subsequent induction of apoptosis in tumor cells. The high membrane permeability of the drug is responsible for its strong bystander effect, which enables it to target HER2-negative cells in addition. In vitro studies demonstrated that SHR-A1811 inhibited tumour growth and could induce regression in a dose-dependent manner. The efficacy of the compound in question was comparable to that of HRA18-C015 (a biosimilar of T-DXd) and anti-HER2-SHR169265 (DAR 8) in various xenograft models [80].

An international phase I study assessed SHR-A1811 for advanced solid tumors that overexpressed or had mutations in HER2 after several treatments [81]. The exposure to the total antibody and SHR-A1811 payload increased with doses ranging from 3.2 to 8.0 mg/kg. The ORR was 61.6% (154 out of 250 patients, 95% CI: 55.3–67.7), including five patients with GC/GEJC. The 6-month PFS rate for all patients was 73.9%. The most prevalent grade 3 or higher AEs were decreased neutrophil count (119 cases, 38.8%) and decreased white blood cell count (70 cases, 22.8%). Furthermore, only 8 patients (2.6%) developed interstitial lung disease.

The SHR-A1811-102 study [82] investigated the potential of SHR-A1811 as a therapeutic agent for the treatment of HER2-expressing locally advanced or metastatic GC/GEJC, as well as colorectal cancer (CRC). As of the data cut-off date of 21 April 2023, preliminary study results showed that 98 patients had been recruited, with 55 of them diagnosed with GC/GEJC. The dose expansion study established that 6.4 mg/kg was RP2D. At the RP2D, the ORRs were 38.2% for GC/GEJC and 43.8% for HER2-positive GC/GEJC, with corresponding sample sizes of 21 out of 55 (95% CI: 25.4–52.3) and 14 out of 32 (95% CI: 26.4–62.3). Additionally, the 6-month PFS was 71.0% ( 95% CI: 54.0-82.7 ) for GC/GEJC and 73.9% for HER2-positive GC/GEJC (95% CI: 52.1–86.9). Among the 98 patients, 67 (68.4%) experienced TRAEs of grade 3 or higher, with hematological toxicities accounting for over 10% of all events. No cases of ILD were reported. A trial is currently underway to evaluate the effectiveness and safety of SHR-A1811 in combination with apatinib for the treatment of advanced GC/GEJC. Furthermore, a phase III clinical trial (NCT06123494) is currently underway to evaluate the efficacy of SHR-A1811 in patients with HER2-positive GC/GEJC who have experienced disease progression following initial anti-HER2 therapy.

### Management of adverse reactions to ADCs 

#### Common adverse reactions

ADCs have been associated with safety concerns, primarily involving hematologic toxicity, gastrointestinal reactions, and fatigue when treating HER2-positive advanced gastric cancer [83, 84]. Hematological toxicity represents a significant problem in the treatment of ADCs, manifesting primarily as neutropenia, anaemia, and thrombocytopenia. The management of these haematological toxicities is typically accomplished through the implementation of dose delays, reductions, and supportive treatments, such as the administration of growth factors or blood transfusions [46, 85]. Common gastrointestinal reactions include nausea, vomiting and decreased appetite. In clinical practice, proactive antiemetic measures, both preventive and therapeutic, should be implemented, along with nutritional support to maintain the patient’s quality of life and treatment compliance. Additionally, fatigue is reported by patients with high frequency, and the aetiology is multifaceted, involving the disease itself, treatment-related toxicity, and psychological factors. Although fatigue is typically of low grade, symptomatic support is necessary, and other potential causes, such as anaemia or thyroid dysfunction, should be ruled out [85]. In general, these common adverse reactions (CARs) are reversible and manageable. Through active monitoring and intervention, it is possible to ensure that patients can tolerate ADCs to a certain extent.

#### Special adverse reactions

Interstitial lung disease (ILD) or pneumonia represents a unique, potentially life-threatening, and severe adverse reaction to ADCs such as T-DXd, requiring special attention from clinicians [58, 86–88]. The cornerstone of ILD/pneumonia management is predicated on the timely identification of the condition and subsequent intervention, given the prevalence of delayed diagnoses [88]. It is imperative that lung imaging (e.g., high-resolution CT) and clinical symptoms (e.g., new or worsening cough, dyspnoea, fever) are monitored regularly during baseline and treatment periods. In instances where ILD/pneumonia is suspected, it is imperative that medication is promptly discontinued. The administration of corticosteroid treatment, such as prednisone or equivalent medications, is essential to regulate the inflammatory response. A range of management strategies has been developed for the treatment of this condition, including the interruption or reduction of doses, the discontinuation of medication, the use of corticosteroids, and the provision of supportive care [87]. The majority of patients are treated by suspending T-DXd administration and using corticosteroids, either in isolation or in combination [89]. The establishment of standardised patient education, monitoring processes, and graded management plans is fundamental to the safe clinical application of ADCs. This encompasses the comprehensive disclosure of the risks and symptoms associated with ILD to patients prior to treatment, the formulation of a meticulous follow-up plan, and the establishment of a collaborative framework involving multidisciplinary teams (comprising oncology, respiratory, and imaging departments) to facilitate the expeditious assessment and management of suspected cases [88]. The implementation of these measures is instrumental in mitigating the fatal risks posed by ILD/pneumonia, thereby ensuring the safety of patient treatment.

In addition, when treating HER2-positive advanced gastric cancer with ADCs, it is imperative to be mindful of other specific adverse reactions, including cardiac toxicity and hepatotoxicity [90]. With regard to cardiac toxicity, a meta‑analysis revealed that T‑DM1 was associated with the lowest incidence of left ventricular ejection fraction (LVEF) decline, while T‑DXd, trastuzumab plus chemotherapy, and trastuzumab plus pertuzumab plus chemotherapy exhibited comparable incidences [91]. In the DESTINY-Gastric01 trial, the decrease in LVEF was not prominently reported as a major cardiac event [38]. Despite being mostly clinically irrelevant in the majority of cases, baseline cardiac assessment, monitoring for cardiac adverse events, and early detection remain essential for HER2-targeted ADCs [92]. Monitoring for hepatotoxicity is also imperative, as it may manifest as elevated transaminases. In clinical trials of T-DXd, liver dysfunction was listed as a common adverse event [90]. Consequently, the regular monitoring of liver function indicators (e.g., ALT and AST) during treatment is imperative to detect and manage liver injury in a timely manner. Moreover, it is imperative to systematically assess and manage other ADC-related toxicities, including peripheral neuropathy and ocular toxicity, throughout the course of long-term treatment [93].

### The mechanisms and countermeasures of drug resistance in ADCs for HER2-positive advanced gastric cancer

ADCs encounter intricate challenges in combating drug resistance in the treatment of HER2-positive advanced gastric cancer, primarily arising from two factors: the target itself and the processes of drug delivery and efficacy. On the one hand, the spatiotemporal heterogeneity of the HER2 antigen has been demonstrated to contribute to resistance [94]. This heterogeneity may result in the downregulation or loss of HER2 expression [95, 96]. Mutations in the HER2 gene itself, such as kinase domain mutations, have been demonstrated to affect antibody binding to the target or downstream signal transduction pathways, thereby leading to resistance. Furthermore, a combined evaluation of HER2 gene amplification in gastric cancer tissues and the expression of proteins such as p53 and Ki67 reveals significant associations between these indicators and neoplasm staging and lymph node metastasis, suggesting that a complex molecular background may jointly influence the response to targeted therapy [41]. It is therefore imperative that a comprehensive understanding of the dynamic changes associated with the target be established in order to overcome ADC resistance.

On the other hand, the efficacy of ADCs is contingent upon the seamless execution of the “targeting-internalization-release” process, wherein any impediment in any phase can result in drug resistance. Firstly, it is important to note that the endocytosis barrier of the ADC-antigen complex, or lysosomal dysfunction, has the capacity to directly affect the release of cytotoxic payloads within the cells [2]. Secondly, the expression of drug efflux pumps, particularly members of the ATP-binding cassette (ABC) transporter family, can pump the effective payload that has entered the cells back out of the cells, significantly reducing its intracellular concentration. This is a common mechanism leading to resistance to payloads such as topoisomerase I inhibitors [97]. Research has demonstrated that the upregulation of ABCG2 and ABCB1 is associated with resistance to T-DXd in models of HER2-positive gastric cancer and lung cancer. This finding indicates that the inhibition of these transporters may restore the sensitivity to ADCs [97].

Furthermore, changes in the action targets of the payload itself or enhanced DNA damage repair capabilities of the cells are also important pathways of resistance. For instance, mutations or downregulation of topoisomerase I (TOP1) and activation of pathways such as homologous recombination repair can render tumor cells insensitive to the cytotoxic effects of ADCs with DXd as the payload (such as T-DXd) [98]. Recent clinical studies have indicated the presence of functionally altered TOP1 mutations in the plasma of metastatic breast cancer patients receiving ADC treatment. These mutations have been linked to a reduction in enzyme activity, a weakening of covalent binding to DNA, and cross-resistance to SN38 and deruxtecan payloads [99]. Collectively, these mechanisms form multiple resistance barriers at the delivery and effect levels of ADCs.

In order to address these challenges, current strategies are focusing on multiple directions. The fundamental approach underpinning this research lies in the development of ADCs that target different epitopes or possess novel properties (e.g., RC48). Concurrently, the exploration of combination therapies involving ADCs and drugs targeting alternative pathways, anti-angiogenic agents, or immune checkpoint inhibitors is intended to circumvent or delay resistance through synergistic effects. The implementation of these multi-faceted strategies is expected to more effectively manage the issue of ADC resistance in HER2-positive advanced gastric cancer.

## Conclusions and prospects

The advent of ADCs has precipitated a paradigm shift in the management of HER2-positive or HER2-low advanced gastric cancer. The advent of a new generation of ADCs, exemplified by T-DXd and RC48, which have demonstrated substantial survival benefits in clinical trials when compared with conventional chemotherapy, has precipitated a substantial transformation in the treatment paradigm for HER2-positive gastric cancer. The DESTINY-Gastric series and the RC48-C008 study have confirmed that ADCs can not only effectively overcome trastuzumab resistance but also demonstrate potential activity in populations with low HER2 expression. This finding suggests that the eligible patient population may further expand.

Nevertheless, the implementation of ADCs in the management of HER2-positive advanced gastric cancer remains encumbered by numerous challenges. Firstly, clinicians must closely monitor drug-related adverse reactions, especially interstitial lung disease/pneumonia and hematological toxicity. Standardised monitoring and management are essential to ensure the safety of treatment. Secondly, the mechanisms of resistance are complex, involving HER2 antigen heterogeneity, endocytic dysfunction, drug efflux pump activation, and target mutations. These factors limit the long-term efficacy of ADCs. Thirdly, significant variations exist in structural design, mechanisms of action, and efficacy and safety among the various ADCs. The failure of T-DM1 in the treatment of gastric cancer suggests that the efficacy of ADCs cannot be simply extrapolated from the treatment of other tumour types and must be accurately assessed based on the unique biological characteristics of gastric cancer.

In the future, the therapeutic application of ADCs for HER2-positive advanced gastric cancer is likely to develop in the following directions: First, the advancement of treatment strategies is underway. Numerous phase III studies are investigating the potential of ADCs in both first-line and second-line treatments, a development expected to reconfigure the prevailing treatment pathways. Second, current research emphasizes the development of combination therapy regimens. The synergistic effects of ADCs with immune checkpoint inhibitors, anti-angiogenic drugs, or other targeted therapies have shown potential in preclinical and early clinical studies. It is reasonable to hypothesize that combination strategies may become critical approaches to overcoming drug resistance and enhance efficacy. Thirdly, the ongoing research and development of novel ADCs is progressively extending the therapeutic window and reducing toxicity by optimising antibody affinity, linker stability, and payload effects. Exemplified by investigational drugs targeting CLDN18.2 (e.g.,SHR-A1904, IBI343) and TROP2 (e.g.,SKB264/Sac-TMT), the systematic and precise design of antibodies, linkers, and payloads enables these agents to achieve enhanced efficacy while offering improved safety, thereby providing new therapeutic opportunities for patients with advanced gastric cancer.

ADCs have become a significant treatment option for HER2-positive or HER2-low advanced gastric cancer. However, further research is required to maximise their clinical value. This should include research to deepen our understanding of the mechanisms of action, to ensure meticulous management of adverse reactions, and to explore combination treatment strategies. The advancement of fundamental research and clinical translation through collaborative efforts has the potential to expand the treatment prospects for HER2-positive advanced gastric cancer.

## Data Availability

No datasets were generated or analysed during the current study.
